# Scale-dependent changes in the functional diversity of macrophytes in subtropical freshwater lakes in south China

**DOI:** 10.1038/s41598-017-08844-8

**Published:** 2017-08-15

**Authors:** Hui Fu, Jiayou Zhong, Shaowen Fang, Jianmin Hu, Chunjing Guo, Qian Lou, Guixiang Yuan, Taotao Dai, Zhongqiang Li, Meng Zhang, Wei Li, Jun Xu, Te Cao

**Affiliations:** 1 Jiangxi Provincial Key Laboratory of Water Resources and Environment of Poyang Lake, Jiangxi Institute of Water Sciences, Nanchang, China; 2 Hydrological Bureau of Jiangxi Province, Nanchang, China; 3 Institute of Hydrobiology, The Chinese Academy of Sciences, Wuhan, 430072 China; 4 Faculty of Resources and Environment, Hubei University, Wuhan, 430062 China; 5 Jiangxi Academy of Environmental Sciences, Nanchang, 330029 China; 6 Institute of Ecology and Environmental Science, Nanchang Institute of Technology, Nanchang, 330099 China

## Abstract

Ecological processes are generally scale-dependent and there is little consensus about the relative importance of deterministic versus stochastic processes in driving patterns of biological diversity. We investigated how the relationship between functional dispersion and environmental gradients changes with spatial scale in subtropical freshwater lakes. The functional alpha and beta dispersions of all the tested traits were significantly under-dispersed across spatial scales and along environmental gradients. Results showed more functional similarity within communities in leaf dry mass content and flowering duration but less functional turnover among communities in all the tested traits at regional scales (Yunnan-Guizhou plateau and the middle and low reaches of the Yangtze River). The strengths and directions of environmental effects on the functional alpha and beta dispersions depended on the selected traits, diversity metrics and spatial scales. Surprisingly, broad-scale factors - elevation and water transparency - decreased the functional turnover for most traits along the gradients, whereas fine-scale factors - water depth - produced the opposite patterns along the gradient, depending on the trait selected. Our study highlights the dominant role of deterministic assembly processes in structuring the local functional composition and governing the spatial functional turnover of macrophyte communities across multiple spatial scales.

## Introduction

The majority of research into biological diversity has focused on determining the factors that drive species richness within a community (taxonomic α-diversity) and species turnover between communities (taxonomic β-diversity), However, species-level analysis of biodiversity are limited, because they treat all species within a community as ecologically equivalent. This ignores the inherent functional variation among species that arise from different life-history strategies within the same environment^1^. For example, a community may have high taxonomic α-diversity, but low functional α-diversity because these species exhibit functional similarity within a given habitat^2^. Similarly, there may be complete species turnover between two communities (i.e. taxonomic β-diversity) but little functional turnover, because these species exhibit functional similarity at different habitats^3^. Therefore, investigating the determinants of functional diversity - which characterizes the physiological dimension of biodiversity omitted by purely taxonomic analyses - will increase our understanding the mechanisms driving the diversity patterns.

Recent analyses have focused on the degree to which the functional diversity of ecological communities is driven by either deterministic process (such as abiotic filtering and biotic competition), or stochastic processes (such as vicariance and random dispersion)^4–8^. These studies have tested the relative importance of deterministic *vs*. stochastic processes in structuring functional diversity using measures of how far the functional α-diversity of each community diverges from a null model^4, 9, 10^. For example, theoretical and empirical analyses suggest that abiotic filtering creates communities with species that are more similar in their functional traits than expected by chance (under-dispersion/convergence). Conversely, biotic interactions are thought to create communities where functional traits are more different than expected by chance (over-dispersion/divergence). Furthermore, recent studies on functional beta diversity have shown that deterministic processes play a more important role in structuring local communities and governing spatial turnover in communities^2, 3, 11–13^. For example, if the co-occurring species in the nearby communities are more functional divergent than the null expectation given the observed species turnover, a higher functional turnover than expected would be detected among communities^2^.

The assembly mechanisms inferred from the observed patterns of functional diversity will be influenced by the spatial scale of analysis^6, 14–19^. This scale-dependency will necessarily affect the results of previous studies that focused on a single spatial scale^20, 21^. A relatively well-accepted hierarchical model of community assembly suggests that environmental factors act as a series of filters (e.g., historical, abiotic, and biotic filters) sequentially sorting species being favorable to local habitats from the regional pool^3, 20, 22^. These hierarchical assembly processes filter species according to sets of functional traits^23^. Firstly, broad-scale environmental factors such as annual rainfall should result in functional similar co-occurring species within large regions (e.g., different river catchments), with little functional turnover among neighboring communities within a large area of homogeneous habitat. Secondly, fine-scale environmental factors such as topography should filter species pool into several subsets with more functionally similar traits along the gradients (e.g., different sites within a river or a lake). A substantial functional turnover between assemblages may exist because of the turnover in the environmental gradient (e.g., water depth gradients within a lake). Finally, the finest-scale (e.g., different microsites within a plot or a depth stratum) is characterized by high individual density and low habitat heterogeneity. With decreasing spatial scales, generally, deterministic processes become less important while stochastic processes become more important in determining community assembly^20^. The scale-dependency of assembly processes are largely attributed to the fact that^17, 20, 24, 25^: (1) the range and extent of environmental filter would change with scale, and (2) the dominant environmental factors would change with scale. For example, elevation or altitude would be important factors driving diversity patterns at regional scale, while local environment (e.g., water depth, nutrients, sediment type) would determine the abundance and distributions of local macrophyte communities^5, 26^. Given the inherent interaction effects of scaling and environment on the assembly processes, few studies have simultaneously focused on the assembly mechanisms along environmental gradients and across multiple spatial scales.

We analyzed how the relationship between functional diversity and environmental gradients changed across four nested spatial scales in south China - 1008 plots, along the water depth gradients within 24 lakes across two regions. We quantified functional alpha and beta dispersion to determine whether the observed functional alpha and beta diversity differs from that expected by chance. We applied a general linear mixed model to decompose the total variance of functional alpha and beta dispersion across spatial scales. Subsequently, we analyzed the significance of environmental effects on functional alpha and beta dispersion at each of three spatial scales (depth, lake and region). We test three questions: First, how do functional alpha and beta dispersion vary across the four spatial scales? Second, how do functional alpha and beta dispersion vary along environmental gradients at each spatial scale? Third, do these diversity patterns related to spatial scale, or environmental gradients differ among functional traits?

## Results

### Effects of spatial scales on functional alpha and beta dispersion

**Figure 1:**
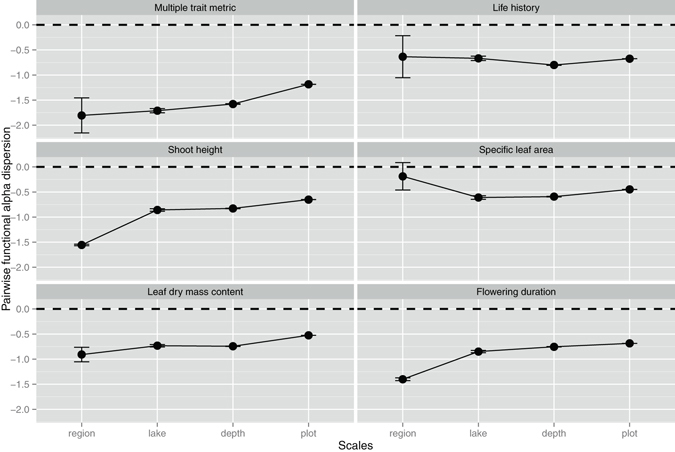
The effects of spatial scale on pairwise functional alpha dispersion. The values lower than 0 indicate a clustering of alpha diversity. The values higher than 0 indicate an over-dispersion of alpha diversity. Each point indicates the mean SES values for each trait metric of each scale.

**Figure 2:**
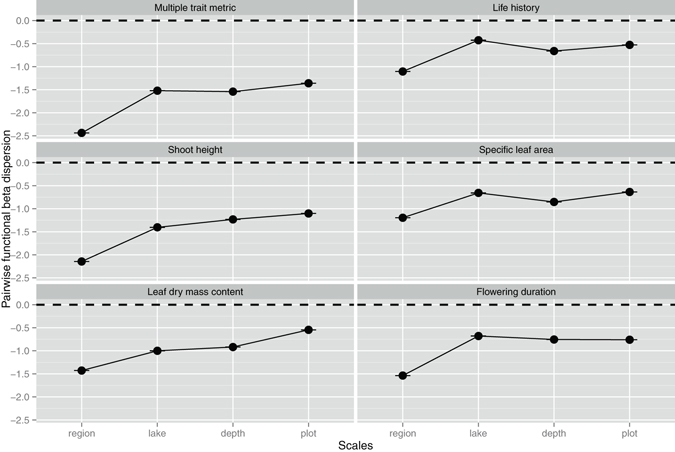
The effects of spatial scale on pairwise functional beta dispersion. The values lower than 0 indicate a clustering of beta diversity. The values higher than 0 indicate an over-dispersion of beta diversity. Each point indicates the mean SES values for each trait metric of each scale.

Functional alpha (SES PW and SES NN) and beta dispersion (SES Dpw and SES Dnn) were quantified using pairwise and nearest-neighbor metrics. Non-random functional alpha and beta dispersion was found for all the traits across all four spatial scales (Table S4, Wilcoxon signed-ranks test, *P* < 0.05). Results showed that functional alpha and beta diversity were significantly clustered (under-dispersion) for multiple trait metric and most individual traits across all four spatial scales (Figs 1 and 2, S3 and S4). The nearest-neighbor functional alpha diversity was significantly over-dispersed for life history at depth and lake scales and for specific leaf area at region scale (Fig. S4).

For both the multi-trait metric and three individual traits – shoot height, leaf dry mass content and flowering duration - pairwise functional alpha diversity showed more clustered patterns (more negative SES values) at broader scales (Fig. 1). For life history and specific leaf area, pairwise functional alpha diversity showed more clustered patterns at finer scales (Fig. 1). Pairwise functional beta diversity exhibited more clustered patterns for multi-trait metric and all individual traits at broader scales (Fig. 2). This result suggests that there was more functional similarity for most traits within communities and less functional turnover among communities at broader scales. For both the multi-trait metric and two individual traits - shoot height and specific leaf area - nearest-neighbor functional alpha diversity showed more clustered patterns at finer scales, while the opposite patterns were noted for leaf dry mass content and flowering duration (Fig. S3). Nearest-neighbor functional beta dispersion showed no clear patterns across spatial scales (Fig. S4).

The partitioning of variance in functional alpha and beta dispersion reveals relatively un-balanced distributions of variance across spatial scales (Fig. S5). Among the four studied spatial scales, plot scale accounted for the most important variations of functional alpha dispersion, ranged from 32.36% (SES PW of flowering duration) to 73.1% (SES NN of life history). Lake scale accounted for the most important variations of functional beta dispersion, ranged from 9.33% (SES Dpw of leaf dry mass content) to 36.04% (SES Dnn of leaf dry mass content). Region scale also accounted for non-negligible variations of functional alpha (0–15.46%) and beta dispersion (0–24.15%). Depth scale accounted for the least variations of functional alpha (0.4–6.36%) and beta dispersion (0–12.36%). Surprisingly, the remained variations of functional alpha and beta dispersion were accounted for by the factors within plot (micro-habitat), ranged from 1.89% (SES PW of life history) to 88.4% (SES NN of shoot height).

### Effects of environmental gradients on functional alpha and beta dispersion

**Figure 3:**
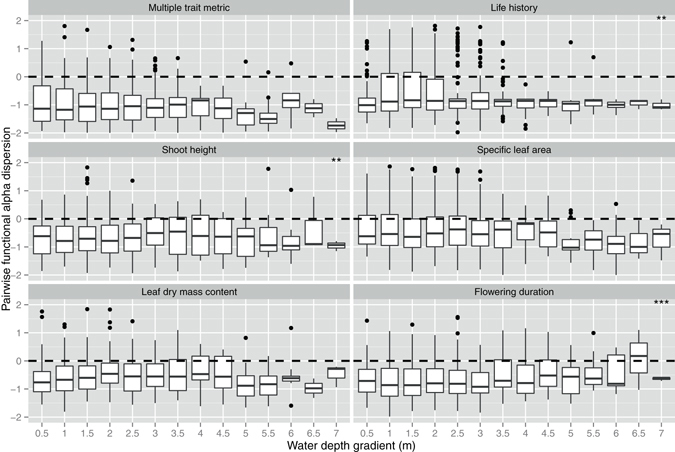
The effects of water depth gradient on pairwise functional alpha dispersion at the depth scale. The values lower than 0 indicate a clustering of alpha diversity. The values higher than 0 indicate an over-dispersion of alpha diversity. *p < 0.05, **p < 0.01, ***p < 0.001. The *p*-values are the results of linear mixed effects models (see Table S5).

**Figure 4:**
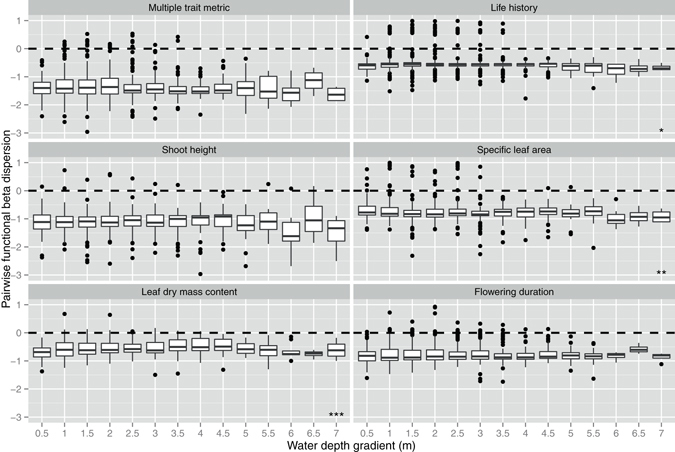
The effects of water depth gradient on pairwise functional beta dispersion at the depth scale. The values lower than 0 indicate a clustering of beta diversity. The values higher than 0 indicate an over-dispersion of beta diversity. *p < 0.05, **p < 0.01, ***p < 0.001. The *p*-values are the results of linear mixed effects models (see Table S5).

At the depth scale, the water depth gradient was considered as the primary environmental factor influencing community dynamics in this study. Significant trends in the SES values of alpha and beta metrics were found along the water depth gradient, although these trends varied in direction, depending upon the metric or traits used (Table S5). Pairwise functional alpha diversity was less clustered for shoot height and flowering duration and more clustered for life history at deeper water (Table S5, Fig. 3). Pairwise functional beta diversity was less clustered for leaf dry mass content and more clustered for life history and specific leaf area toward deeper water (Table S5, Fig. 4). Nearest-neighbor functional alpha diversity was less clustered for shoot height and flowering duration at deeper water, and more clustered for life history and specific leaf area at deeper water (Table S6, Fig. S6). Nearest-neighbor functional beta diversity generally became less clustered for all individual traits and multi-trait metric along the water depth gradient (Table S6, Fig. S7).

**Figure 5:**
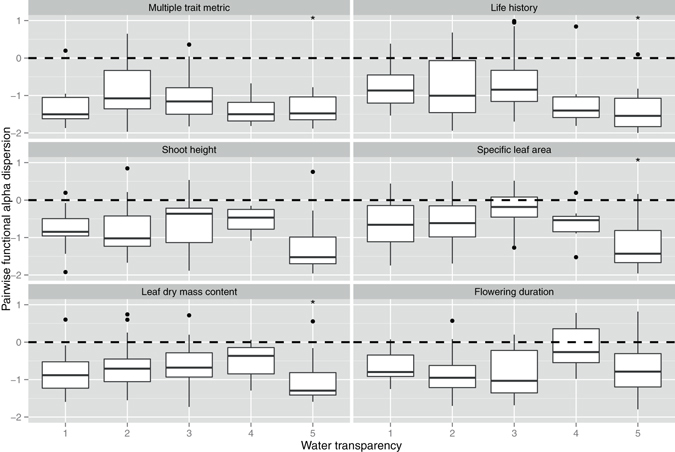
The effects of water transparency (Secchi depth, SD) on pairwise functional alpha dispersion at the lake scale. The values lower than 0 indicate a clustering of alpha diversity. The values higher values than 0 indicate an over-dispersion of alpha diversity. We separated the log-transformed SD gradients as five levels (1: 1.47–1.76; 2: 1.76–2.06; 3: 2.06–2.36; 4: 2.36–2.66; 5: 2.66–2.95). *p < 0.05, **p < 0.01, ***p < 0.001.The *p*-values are the results of linear mixed effects models (see Table S5).

**Figure 6:**
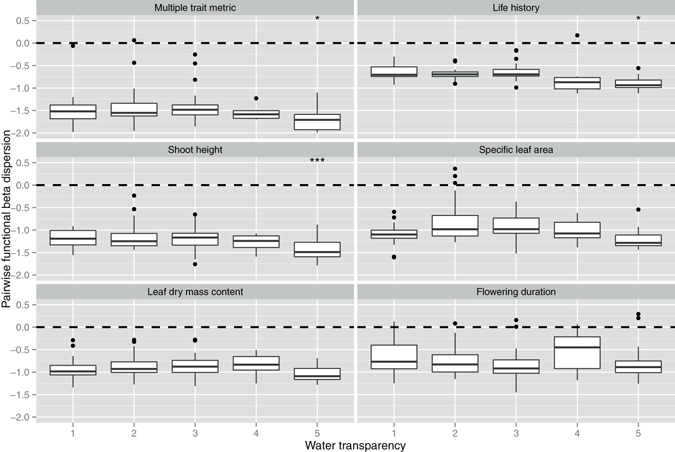
The effects of water transparency (Secchi depth, SD) on pairwise functional beta dispersion at the lake scale. The values lower than 0 indicate a clustering of beta diversity. The values higher than 0 indicate an over-dispersion of beta diversity. We separated the log-transformed SD gradients as five levels (1: 1.47–1.76; 2: 1.76–2.06; 3: 2.06–2.36; 4: 2.36–2.66; 5: 2.66–2.95). *p < 0.05, **p < 0.01, ***p < 0.001. The *p*-values are the results of linear mixed effects models (see Table S5).

At the lake scale, the water transparency (Secchi depth in this study) was the most important environmental factor according to PCA from the six measured physical and chemical parameters of water. Pairwise functional alpha diversity was more clustered for multi-trait metric and three individual traits - life history, specific leaf area and leaf dry mass content - at lakes with higher transparency (Table S5, Fig. 5). Pairwise functional beta diversity became more clustered for multi-trait metric and two individual traits - life history and shoot height - at lakes with higher transparency (Table S5, Fig. 6). Nearest-neighbor functional alpha dispersion showed no significant trends for either multi-trait or individual trait metrics in relation to water transparency (Table S6, Fig. S8). Nearest-neighbor functional beta diversity became more clustered for multi-trait metric and all individual traits at lakes with higher transparency (Table S6, Fig. S9).

**Figure 7:**
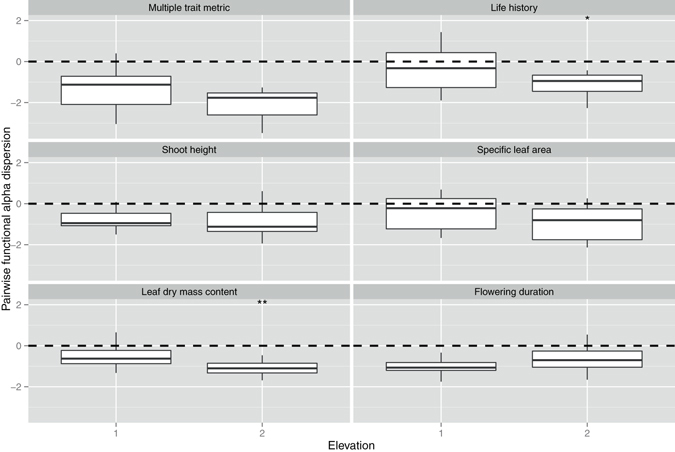
The effects of elevation (1, lakes with low elevation at the middle and lower reaches of the Yangtze River, 16.3 m above sea level; 2, lakes with high elevation at Yunnan-guizhou plateau, 1967.4 m above sea level) on pairwise functional alpha dispersion at the regional scale. The values lower than 0 indicate a clustering of alpha diversity. The values higher than 0 indicate an over-dispersion of alpha diversity. *p < 0.05, **p < 0.01, ***p < 0.001. The *p*-values are the results of linear mixed effects models (see Table S5).

**Figure 8:**
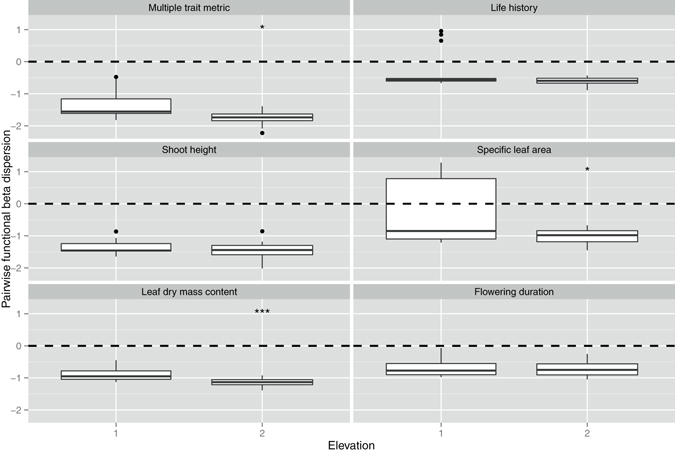
The effects of elevation (1, lakes with low elevation at the middle and lower reaches of the Yangtze River, 16.3 m above sea level; 2, lakes with high elevation at Yunnan-guizhou plateau, 1967.4 m above sea level) on pairwise functional beta dispersion at the regional scale. The values lower than 0 indicate a clustering of beta diversity. The values higher than 0 indicate an over-dispersion of beta diversity. *p < 0.05, **p < 0.01, ***p < 0.001. The *p*-values are the results of linear mixed effects models (see Table S5).

At the regional scale, elevation was considered as the major environmental factor influencing community dynamics in this study. Pairwise functional alpha dispersion was more clustered for life history and leaf dry mass content at higher elevation (Table S5, Fig. 7). Pairwise functional beta dispersion was more clustered for multi-trait metric and two individual traits -specific leaf area and leaf dry mass content - at higher elevation (Table S5, Fig. 8). Nearest-neighbor functional alpha dispersion was more clustered for leaf dry mass content and less clustered for flowering duration at higher elevation (Table S6, Fig. S10). Nearest-neighbor functional beta dispersion showed no significant functional turnover for either multi-trait or individual trait metrics in relation to elevation (Table S6, Fig. S11).

## Discussion

Here we quantified the functional alpha and beta diversity of macrophyte communities along environmental gradients at multiple spatial scales in 24 freshwater lakes of south China. We found that the patterns of functional diversity within community as well as functional turnover among communities were strongly nonrandom across all four spatial scales. Furthermore, the strength of nonrandom effects on the functional composition of macrophyte significantly varied with spatial scale and along the environmental gradients at each spatial scale. Our results provide strong support for the deterministic processes that drive macrophyte community assembly from the local to regional scales.

The first goal of this study was to estimate how functional alpha and beta dispersion vary across spatial scales, given the observed species alpha and beta diversity. Our results showed that the observed functional alpha and beta diversities were generally lower than that the null expectation given the species richness and turnover, as indicated by the significant clustering of functional alpha and beta dispersion in most cases. This result indicated that niche-based deterministic processes are relatively more important than stochastic processes in structuring the macrophyte community across spatial scales, which is consistent with previous studies on terrestrial plant communities^2, 3, 11, 12, 27^. Variations of the functional alpha dispersion across spatial scales largely depend on the selected traits. For example, the strengths of abiotic filtering effects on functional diversity within a community were relatively lower in some functional axes (i.e., leaf dry mass content and flowering duration) but higher in other functional axes (i.e., life history and specific leaf area) with decreasing spatial scale. This result suggests that deterministic assembly processes shape the functional composition of local community in multidimensional functional space, and are strongly related to the trade-offs among functional traits (e.g., leaf dry mass content *vs*. specific leaf area)^5, 9, 10, 28^. By contrast, the variations in functional beta dispersion across spatial scales were highly consistent for the metrics of multi-trait and all individual traits. The strength of abiotic filtering effects on functional turnover among communities was shown to be lower at finer spatial scales. This study highlighted that there are generally more functionally similar co-occurring species within community with less functional turnover among communities at broader scales.

Variance partitioning of functional dispersion demonstrated that the spatial structure of total variance was largely dependent on scale and trait metrics. This result suggests that environmental gradients in different scales may have different roles in shaping functional diversity patterns across spatial scales. As previous study suggested^29^, we can decompose the studied spatial scales into external (region, lake and depth scales) and internal (plot and within plot scale) filtering processes. The present study demonstrated that the functional diversity patterns across spatial scales for multiple trait metrics and individual traits were greatly affected by internal filtering processes (>50%), implying that the environmental heterogeneity in micro-habitat and biotic interactions were highly important in determining functional compositions of macrophyte community. Overall, regional and lake scale explained more variations of functional beta dispersion, while plot scale accounted for more variations of functional alpha dispersion.

The second goal of this study was to estimate how functional alpha and beta dispersion vary along environmental gradients at specific spatial scales, given the observed species alpha and beta diversity. In the present study, we focused on one major environmental gradient for each of three spatial scales, namely, elevation at the regional scale, water transparency at the lake scale and water depth at the depth scale. Study results showed that the observed functional alpha and beta diversities were generally lower than the null expectations along the three environmental gradients given the species richness and turnover, as indicated by the significant clustering of functional alpha and beta dispersion in most cases. This result firstly demonstrated that there were significant deterministic assembly processes that structure the functional composition of macrophyte communities along each of the three environmental gradients across three nested spatial scales. Several previous studies on the mechanisms of community assembly have focused on a specific scale, although deterministic assembly has been observed in terrestrial^10, 30, 31^, freshwater^5, 32^ and marsh plant communities^33^, even in tropical forests^4, 9^ that may be driven by neutral assembly processes. Freshwater macrophytes generally show broad distributional ranges from the local to regional scales, which is largely attributed to their high trait plasticity (i.e., functional diversity) in various freshwater habitats^34^. Freshwater habitats represent a stressful environment for plants, characterized by low carbon availability, shaded conditions, sediment anoxia, mechanical damage by currents and waves and a limited nutrient supply^35^. Furthermore, the primary environmental factors influencing macrophyte communities would change from broad scales (i.e., water chemistry and nutrient supply among lakes) to fine scales (i.e., water depth, slope and sediment characteristics among sites)^26, 29^. According to the hierarchical assembly processes, the three nested environmental gradients (i.e., elevation, water transparency and water depth from regional to local scales) in the present study should have sequentially filtered species being favorable to local habitats from the regional species pool.

Surprisingly, the strengths and directions of environmental effects depend on the selected traits, diversity metrics and spatial scales. For example, macrophyte species tend to be more functionally similar within communities for life history at deeper water (depth scale), lakes with higher transparency (lake scale) and regions with higher elevation (regional scale), while be less functionally similar for shoot height at deeper water. This result suggests that abiotic filtering effects increased for life history but decreased for shoot height along water depth gradients, associated with the intrinsic trade-offs among different sets of traits along the gradients. On the other hand, functional beta dispersions were generally lower than the null expectations across scales; nearby communities increased functional turnover - less departure from the null expectation - for leaf dry mass content along the water depth gradient, while decreased functional turnover for life history and specific leaf area toward deep waters. With increased water transparency and elevation, however, nearby communities decreased functional turnover for multi-trait metric and two individual traits - specific leaf area and leaf dry mass content. Results indicated that broad-scale environmental factors - elevation and water transparency - resulted in decreased functional turnover for most traits along the gradients, and fine-scale environmental factors - water depth - lead to two completely opposite patterns along the gradient, depending on the trait selected. In this study, the regions with higher elevation in the Yunnan-Guizhou plateau and the lakes with higher water transparency are relatively favorable to the growth of macrophytes. The former showed a longer growth period, higher light radiation intensity and constant annual temperature, while the latter had a higher light availability^29^. Abiotic filtering effects are usually weakened when environmental conditions become favorable for plants at broad scales; thus, nearby communities should become more functionally similar. For this point, less functional turnover was observed in benign habitats than in adverse ones at the two scales (i.e., region and lake). However, the trait distribution at the local scale should be shaped by the hierarchical effects of abiotic and biotic filters^1, 10, 33^. Our recent study demonstrated that niche differentiation (i.e., biotic filters) has a structuring role in the macrophyte community assembly along the water depth gradient; the effects of abiotic and biotic filters may act strongly on some traits but weakly on others, linking the intrinsic trade-offs among different sets of traits along the gradient^5^. Thus, the combined effects of abiotic and biotic filters may be responsible for the opposite patterns of functional turnover among the tested traits along the water depth gradient.

However, we also found no significant effects of environmental gradients - water depth, water transparency and elevation - on functional alpha and beta dispersions for several tested traits, thereby implying that the strength of deterministic processes in these functional axes did not vary with environmental gradients. Furthermore, no significant biotic effects (i.e., functional over-dispersion) on the functional composition of macrophyte communities were found in most tested traits of this study. Fu, *et al*.^5^ indicated that biotic interactions played a structuring role on macrophyte community assembly at local scale, and that including intraspecific trait variability into functional alpha diversity greatly enhanced the detections of non-random biotic effects. Spasojevic, *et al*.^36^ highlighted that the integration of intraspecific trait variability into functional beta diversity would better explain the diversity patterns across spatial scales. The present study measured the metrics of functional alpha and beta diversity based on the interspecific trait variability, and thus important information might have been lost from intraspecific trait variability^18, 37^.

To our knowledge, this study is the first to focus on the effects of spatial scale on the functional alpha and beta dispersion for aquatic macrophytes. Results provide fresh insights into the mechanisms that drive macrophyte diversity patterns across spatial scales and in relation to environmental gradients. Overall, the present study leads to three important conclusions. Firstly, the functional alpha and beta dispersion of most traits was clustered across all four spatial scales, thereby highlighting the dominant role of deterministic processes at the scales of this study. Secondly, the effects of spatial scale on functional alpha dispersion depended on the selected traits; the strength of abiotic filtering effects increased for some traits but decreased for others as the spatial scale increased. For functional beta dispersion, the strength of abiotic filtering effects largely decreased (i.e., less clustered) from the regional to local scale. Finally, the strengths and directions of environmental effects on the functional alpha and beta dispersions depended on the selected traits, diversity metrics and spatial scales. Surprisingly, broad-scale abiotic factors - elevation and water transparency - resulted in decreased functional turnover for most traits along the gradients. Fine-scale abiotic factors - water depth - produced two completely opposite patterns along the gradient, depending on the trait selected. Overall, the deterministic assembly processes would drive functional diversity patterns by shaping the multi-dimensional functional space, which is constructed from functional traits representing major axes of plant strategy.

## Methods

### Field sampling

Field surveys of macrophyte communities were conducted in 11 lakes on the Yunnan-Guizhou plateau in China, during June to August 2014 (see Fig. S1). Further surveys were conducted in 13 lakes along the middle and lower reaches of the Yangtze River in China, during June to August 2015 (see Fig. S1. and Table S1 for the physical and chemical properties of these lakes). The abundance of the macrophyte community was estimated in a series of 25 m^2^ plots in 3 (in a smaller lake)-45 (in a larger lake) sites of these lakes (Fig. S1). The final data set consisted of 1008 plots. The sites were selected to represent the full range of the water depth gradient and macrophyte community variation along this gradient. At each site, a series of 5 m × 5 m plots were located along the water depth gradient in each 0.5 m interval as a depth stratum. These surveys extended from the marginal to pelagic regions, where the water depth varied from 0.5 m to 7 m and depended on the maximum colonization depth of macrophyte at specific sites of the lakes. For each lake, 2 (for the shallower lakes)-14 (for the deeper lakes) water depth strata were included. The plots were randomly assigned to the water depth gradients in areas dominated by macrophyte species. Locations that were disturbed by recent human activity (e.g., mowing and fishing) were excluded from sampling. Three 0.2 m^2^ quadrats of macrophyte were sampled within each 25 m^2^ plot by a rotatable reaping hook (diameter = 0.5 m, area = 0.2 m^2^). All the species in these plots were identified and recorded. Fifty species including 27 submerged macrophytes, 12 floating-leaved macrophytes and 11 emergent macrophytes were recorded in the 1008 sampling plots along the water depth gradients of 24 lakes across 2 regions (Table S1). The mean trait values of each species are shown in Table S2. Species richness ranged from 1 to 18 species per plot with the median plot containing 4 species.

### Functional trait measurements

Five key functional traits (shoot height, specific leaf area and leaf dry mass content)were measured, and two traits (life history and flowering duration) were collected from the literature for the 50 macrophyte species following standardised protocols^38^: life history (annual and perennial), shoot height, specific leaf area, leaf dry mass content and flowering duration. The life history and flowering duration for each species were collected from regional floras. Flowering duration describe the earliest and latest months that a species is in flower. The details of the trait measurements were described in Fu *et al*.^5, 39^.

We measured the mean values of the following three functional traits from 50 individuals of each species: shoot height, specific leaf area, leaf dry mass content. Specific leaf area is part of the leaf economic spectrum and is closely correlated with photosynthetic capacity, nitrogen content per mass and leaf life span^40, 41^. Leaf dry mass content reflects the fundamental trade-off in investing resources in structural tissues vs. liquid-phase processes and therefore has been argued to be the root variable that governs the correlations among the traits in the leaf economic spectrum^24, 40–42^. Shoot height (cm) is often allometrically related to overall plant size and competitive interactions for light^43^.

### Spatial scales and environmental gradients

To test the effects of spatial scale on functional alpha and beta diversity, the diversity metrics were calculated at four hierarchical nested spatial scales: plot scale (*n* = 1008), depth scale (*n* = 135), lake scale (*n* = 24), and regional scale (*n* = 2). The plots were nested within different depth strata, the depth strata were nested within different lakes, and the lakes were nested within different region. We defined ecological communities at four nested spatial scales. Firstly, the plot scale was defined as four quadrats pooled into a 25 m^2^ plot (Fig. S2a). Secondly, the depth scale was defined as all 25 m^2^ plots at the same water depth (Fig. S2b). Third, the lake scale was defined as all depths within the same lake Fig. S2c). Fourth, the regional scale was defined as all lakes within one of two regions (Fig. S2d).

We assessed how functional alpha and beta diversity varied along the environmental gradients on at three spatial scales -depth, lake and regional scales. At the depth scale, the water depth gradient was the major factor influencing community diversity. At the lake scale, a principal components analysis (PCA) was used to extract orthogonal axes of lake from the six measured physical and chemical parameters of water (Secchi depth, total nitrogen content, total phosphate content, chlorophyll-a concentration, PH and water temperature during study period) and to reduce information redundancy (Table S3). Prior to PCA, the six parameters were log-transformed and standardized with a mean of 0 and standard deviation of 1. The first principal components accounted for over 58% of the variation. The first PCA axis was positively correlated with Secchi depth (hereafter called water transparency) and negatively correlated with total nitrogen content, total phosphate content and chlorophyll-a concentration. The first PCA axis suggested that higher water transparency was associated with lower nutrient conditions and lower phytoplankton abundance in lakes.. Therefore, we included water transparency as the primary environmental factor for subsequent analyses at the lake scale. We separated evenly the log-transformed water transparency as five levels (1: 1.47–1.76; 2: 1.76–2.06; 3: 2.06–2.36; 4: 2.36–2.66; 5: 2.66–2.95). At the regional scale, the lakes of Yunnan-Guizhou plateau (1967.4 m above sea level) had a significantly higher average elevation than those of the middle and lower reaches of the Yangtze River (16.3 m above sea level). Thus, we included elevation as a primary environmental factor for subsequent analyses at the regional scale.

### Functional alpha and beta diversity metrics

Functional alpha and beta diversities of macrophyte communities were calculated using two dendrogram-based metrics: mean pairwise and nearest-neighbor trait distance, respectively^44^. The distances between individuals were calculated from a trait dendrogram, which is the mean distance of all species to the weighted centroid of the community in the trait space^44^. The trait dendrogram was generated by first generating a trait Euclidean distance matrix for the continuous data, and a trait Gower distance matrix for the discrete or mixed data (i.e., multi-trait metric). Hierarchical clustering was then applied to these matrices to generate the dendrogram. We generated six trait dendrograms, including ten for each trait (single trait) and one for all the traits combined (multi-trait metric).

To calculate the functional alpha diversity, the mean pairwise trait distance (PW) and nearest-neighbor trait distance (NN) were weighted by abundance and quantified from the functional trait dendrogram for all the individuals in a community (Fig. S2)^44^.

To quantify the functional beta diversity, two abundance weighted metrics were measured according the approach of Swenson, *et al*.^2^. The first metric calculated the mean pairwise functional dissimilarity (Dpw) between two communities. The second metric calculated the mean nearest functional neighbor dissimilarity (Dnn) between two communities. We used these two metrics of functional beta diversity because they represent the two main mathematically independent classes of functional dissimilarity metrics, which avoid the redundancy of calculating other identical metrics^44^.

To quantify the functional beta diversity (the turnover in functional traits between communities) within each depth stratum (*n* = 135), we pooled all quadrats within each plot, and calculated functional dissimilarity among all plots at each depth stratum of each lake (Fig. S2). To quantify the functional beta diversity within each lake (*n* = 24), we pooled all depth stratum within each lake and calculated the functional dissimilarity among the depth strata of each lake. To quantify the functional beta diversity within each region (*n* = 2), we all pooled all lakes within each region and calculated the functional dissimilarity among the lakes of each region. To measure the functional beta diversity among both regions, we calculated functional dissimilarity among the two studied regions.

### Null models

At each spatial scale, we tested whether the observed functional alpha and beta diversity metrics differed from those expected by chance, using a null model. The null model was generated by randomly assigning the taxa names to the tips of the trait dendrogram for 999 times^44^, and calculating functional alpha and beta diversity for ach iteration. The species alpha and beta diversity, species occupancy rates, abundances and spatial distributions were fixed for each iteration^3, 44^. If functional diversity values were lower than expected by chance, this indicated functional clustering (under-dispersion) within communities for alpha diversity, and functional clustering among communities for beta diversity. If functional diversity values were higher than expected by chance, this indicated a functional over-dispersion within communities for alpha diversity, and a functional over-dispersion among communities for beta diversity.

We used a standardized effect size (SES) to assess the standard deviation of the observed trait distribution from the null trait distribution^45^ (equation 1):

1 $${\rm{SES}}=({{\rm{Metric}}}_{{\rm{obs}}}-{{\rm{Metric}}}_{{\rm{null}}})/{{\rm{Metric}}}_{{\rm{SD}}}$$

where Metric_obs_ is the value of the observed metric in a community at each scale, Metric_null_ is the mean value of the metric for quadrats in 999 null communities and Metric_SD_ is the standard deviation of the metric for quadrats in 999 null communities^45^. SES quantifies the direction and magnitude of the deviation of each plot from the null distribution. A negative SES value indicates that the traits are under-dispersed in a community for each scale, whereas a positive SES value indicates that the traits are over-dispersed in a community for each scale. We applied a Wilcoxon signed-rank test to examine whether the mean values of SES were significantly different from their null expectations zero for each spatial scale^46^.

To test how functional alpha (i.e., SES PW and SES NN) and beta dispersions (i.e., SES Dpw and SES Dnn) varied with spatial scales, we fitted a general linear mixed model to the total variance of functional alpha and beta dispersion across four scales nested one into another (i.e. nested ANOVA with random effects) in this increasing order: plot, depth, lake and region. A variance component analysis was performed on this model using the ‘varcomp’ function of R^47^.

To test how functional alpha and beta dispersion varied with environmental gradient of each spatial scale, a linear mixed effects model was generated for the three spatial scales (i.e., depth, lake and regional scales), with the SES values of each metric for each scale as the dependent variables. To quantify the optimal structure of fixed-effect and random-effect in linear mixed effect models, we followed the top-down approach described by Zuur, *et al*.^48^, which contains four major steps: (1) identify the optimal error structure (using AIC_c_); (2) identify the optimal fixed-effect structure for the given random-effect structure through likelihood ratio tests; (3) check the final model assumptions (heterogeneity, normality, and independence of residuals); (4) present the final model using restricted maximum likelihood estimation (REML) as the likelihood estimator, as it is considered to be a less biased estimator^48^. The *P*-values of the models were obtained by the likelihood ratio test. If the SES values of a trait metric become more negative than the null expectation along the gradient, we can expect a stronger effect of abiotic filtering effects on this trait; if the SES values of a trait metric become more positive than the null expectation along the gradient, we can expect a stronger effect of biotic filtering effects on this trait.

All analyses were performed in R using the packages “vegan”, “picante”, “coin” and “lme4”^47, 49–51^. The figures were obtained using ArcGis 10.0 and the R packages “ggplot2”^52, 53^.

## Electronic supplementary material

Supplementary Information
