# Supplementary material for: Scale-dependent changes in the functional diversity of macrophytes in subtropical freshwater lakes in south China
Source: Sci Rep. 2017 Aug 15;7:8294. doi: 10.1038/s41598-017-08844-8 (PMC5557923; doi:10.1038/s41598-017-08844-8)
Supplement: Supplementary file 1 — Supplementary Information [file 41598_2017_8844_MOESM1_ESM.pdf]

Scale-dependent changes in the functional diversity of macrophytes in subtropical freshwater lakes in south China

Hui Fu[1*], Jiayou Zhong[1], Shaowen Fang[2], Jianmin Hu[1], Qian Lou[1], Chunjing Guo[1], Guixiang Yuan[1], Taotao Dai[1], Zhongqiang Li[4], Meng Zhang[5], Wei Li[6], Jun Xu[3], Te Cao[3]

[1]Jiangxi Provincial Key Laboratory of Water Resources and Environment of Poyang Lake, Jiangxi Institute of Water Sciences, Nanchang, China

[2]Hydrological Bureau of Jiangxi Province, Nanchang, China

[3]Institute of Hydrobiology, The Chinese Academy of Sciences, Wuhan 430072, China

[4]Faculty of Resources and Environment, Hubei University, Wuhan 430062, China

[5]Jiangxi Academy of Environmental Sciences, Nanchang 330029, China

[6]Institute of Ecology and Environmental Science, Nanchang Institute of Technology, Nanchang 330099, China

*** Corresponding author.** Email: huifu367@163.com

Lake	Region	Elevation (a.s.l. m)	Surface area (km^2)	Storage capability ($\times10^8 m^3$)	Average water depth (m)	Maximum water depth (m)	SD	TN	TP	Chl. a	pH	WT
Bo Lake	1	14	238	6.9	2.60	5.40	65	0.729	0.039	71.660	7.40	27.50
Changdang Lake	1	9	90	1.1	1.22	2.88	35	1.205	0.106	50.250	7.96	25.00
East Tai Lake	1	9	2428	51.5	1.20	3.34	114	0.675	0.020	6.248	8.58	30.42
Gucheng Lake	1	8	31	0.4	1.56	4.40	107	0.960	0.067	20.120	8.10	28.10
Gaoyou Lake	1	6	780	4.2	1.92	4.50	70	0.743	0.038	24.502	8.60	28.08
Huangda Lake	1	14	297	8.0	4.00	5.00	160	1.160	0.020	78.260	7.50	21.00
Hong Lake	1	25	348	4.7	1.35	2.32	84	0.763	0.065	32.518	7.66	26.67
Futou Lake	1	21	130	3.3	2.90	4.40	82	1.171	0.049	14.165	7.75	25.77
Hongze Lake	1	13	1950	42.0	1.60	3.60	29.3	2.240	0.156	68.560	7.80	26.21
Liangzi Lake	1	19	304	10.8	2.25	4.80	115	0.661	0.039	26.067	8.30	31.39
South Dongting Lake	1	34	2820	188.0	6.39	23.50	39	0.566	0.016	8.620	7.40	27.89
Wuchang Lake	1	11	87	0.4	2.20	2.47	118	0.599	0.018	13.225	7.20	25.95
Xiliang Lake	1	21	80	1.4	1.94	2.60	191	0.339	0.016	3.853	10.06	27.12
Caohai Lake	2	2172	25	0.6	2.40	5.00	120	0.319	0.017	15.200	8.22	18.25
Chenghai Lake	2	1503	77	19.9	25.70	35.10	137	0.101	0.034	26.784	9.20	24.70
Dianchi Lake	2	1886	298	11.7	2.93	5.87	54	0.202	0.106	81.782	8.62	23.25
Erhai Lake	2	1974	250	25.4	10.80	20.70	180	0.527	0.088	17.639	8.60	23.61
Fuxian Lake	2	1722	217	189.0	89.60	157.30	544	0.027	0.008	3.286	8.80	23.89

Jian Lake	2	2184	6	0.3	2.70	6.00	260	0.395	0.011	5.260	7.60	23.65
Lugu Lake	2	2691	50	22.2	40.30	105.30	898	0.028	0.010	1.008	8.55	21.36
Lashihai Lake	2	2440	9	0.2	2.10	4.30	133	0.091	0.034	9.486	7.50	19.25
Qilu Lake	2	1790	42	1.5	4.00	6.80	55	0.622	0.081	55.140	9.50	24.96
Xingyun Lake	2	1722	39	1.8	7.00	10.00	34	0.601	0.797	437.472	8.90	24.28
Yangzonghai Lake	2	1768	31	6.0	20.00	30.00	103	0.078	0.032	25.389	8.20	25.33

Table S1 The selected morphometrical and limnological characteristics in 24 freshwater lakes at two regions (1, lakes with low elevation at the middle and lower reaches of the Yangtze River; 2, lakes with high elevation at Yunnan-guizhou plateau). SD: Secchi depth (cm), TN: total nitrogen content in water (mg L^{-1}), TP: total phosphate content in water (mg L^{-1}), Chl. a: chlorophyll-a concentration (mg L^{-1}), WT: water temperature (℃) during study period.

Species	LH	SH	SLA	LDMC	LT
Alisma plantago-aquatica	2	38.246	31.3852	140.008	0.25917
Alternanthera philoxeroides	2	16.2115	27.8069	125.979	0.25333
Batrachium bungei	2	42.541	68.1351	121.243	0.0964
Cardamine lyrata	2	5.03448	53.0232	173.651	0.15
Ceratophyllum demersum	2	95.5385	83.9277	81.3974	0.50651
Eichhornia crassipes	2	21.12	26.1835	114.882	0.32327
Elodea densa	2	36.45	98.1553	102.221	0.1324
Elodea nuttallii	1	86.54	106.2	69.374	0.1536
Euryale ferox	1	89.211	28.7655	142.065	0.29143
Heleocharis dulcis	2	30.3333	3.51986	304.08	1.39333
Hydrilla verticillata	2	106.566	118.952	163.855	0.10237
Hydrocharis dubia	1	105.26	31.7433	91.6167	0.456
Limnophila sessiliflora	2	53.14	96.213	54.2183	0.18
Monochoria vaginalis	1	19.214	39.4651	113.086	0.2825
Myriophyllum spicatum	2	90.0216	109.61	138.704	0.29007
Najas graminea Del.	1	56.82	62.21	55.0873	0.775
Najas marina	1	45.6	90.5227	73.7504	0.42333
Najas minor	1	42.54	63.12	57.0004	0.22
Najas orientalis	1	55.62	65.12	57.7434	0.12
Nelumbo nucifera	2	112.25	25.1289	196.246	0.35056
Nymphoides indica	2	72.536	27.576	106.574	0.51667
Nymphoides peltatum	2	111.961	46.4032	143.823	0.47
Ottelia acuminata	2	135.21	102.777	70.1745	0.1242
Ottelia alismoides	1	66.243	103.041	48.2656	0.35
Phragmites australis	2	118	15.4623	350.377	0.16119
Polygonum amphibium	2	152.167	44.9863	238.742	0.21375
Polygonum hydropiper	1	25.1667	44.0469	228.502	0.245
Potamogeton acutifolius	2	86.465	87.628	195.111	0.1324
Potamogeton crispus	2	125.61	99.2135	101.013	0.1539
Potamogeton distinctus	2	80.6667	58.5321	179.899	0.25765
Potamogeton intortifolius	2	149.068	75.1189	210.291	0.10525
Potamogeton lucens	2	163.82	87.2725	195.197	0.11635
Potamogeton maackianus	2	150.75	121.529	198.246	0.14842
Potamogeton malaianus	2	151.458	89.3554	200.687	0.11929

Potamogeton natans	2	123.54	42.9536	236.268	0.1956
Potamogeton oxyphyllus	2	68.34	41.524	196.223	0.1534
Potamogeton pectinatus	2	132.722	82.2612	143.189	0.44884
Potamogeton perfoliatus	2	116.907	93.9877	104.346	0.11816
Potamogeton praelongus	2	58.6667	146.495	153.846	0.09
Potamogeton pusillus	2	75.244	92.534	115.671	0.1034
Rumex japonicus	2	12.2778	66.0038	105.112	0.33
Trapa litwinowii V. Vassil.	1	126.25	15.2746	224.862	0.24
Trapa natans	1	147.685	30.7556	235.809	0.40817
Triarrhena sacchariflora	2	48.4341	19.7166	377.243	0.1375
Typha orientalis	2	125.213	11.3366	291.724	1.26389
Utricularia aurea Lour.	1	35.15	106.451	46.1818	0.09342
Vallisneria denseserrulata	2	53.24	55.9548	59.0504	0.55563
Vallisneria natans	2	92.444	73.2043	84.7276	0.63775
Zannichellia palustris	2	18.2446	56.3465	211.243	0.1048
Zizania latifolia	2	98.8542	18.6482	348.891	0.16387

Table S2 The mean values of 10 traits for the 50 recorded macrophyte species in the studied areas. LH indicates life history (1-annual, 2- perennial); SH indicates shoot height (cm); SLA indicates specific leaf area (cm^2 g^{-1}); LDMC indicates leaf dry mass content (g g^{-1}); FD indicates flowering duration.

Environmental factors	Axis 1	Axis 2	Axis 3
Secchi depth (cm)	0.511	0	0
Total nitrogen content in water (mg L^{-1})	-0.458	-0.243	0.179
Total phosphate content in water (mg L^{-1})	-0.45	0.332	-0.265
Chlorophyll-a concentration (mg L^{-1})	-0.486	0.187	-0.242
pH	0.151	0.888	0.277
Water temperature (℃) during study period	-0.243	0	0.873
% variation	58.42	17.4	16.4

Table S3 The principle component analysis (PCA) on the six environmental factors across 24 studied lakes.

	SES PW			SES NN			SES Dpw			SES Dnn		
	Mean	SE	*P*	Mean	SE	*P*	Mean	SE	*P*	Mean	SE	*P*
Plot scale												
multi.FD	-1.185	0.001	**<0.001**	-1.007	0.001	**<0.001**	-1.36	0.000	**<0.001**	-2.495	0.000	**<0.001**
LH	-0.674	0.001	**<0.001**	-0.183	0.001	**<0.001**	-0.527	0.000	**<0.001**	-1.760	0.000	**<0.001**
SH	-0.651	0.001	**<0.001**	-0.62	0.001	**<0.001**	-1.103	0.000	**<0.001**	-2.014	0.000	**<0.001**
SLA	-0.449	0.001	**<0.001**	-0.315	0.001	**<0.001**	-0.635	0.000	**<0.001**	-1.930	0.000	**<0.001**
LDMC	-0.525	0.001	**<0.001**	-0.493	0.001	**<0.001**	-0.545	0.000	**<0.001**	-1.814	0.000	**<0.001**
FD	-0.686	0.001	**<0.001**	-0.536	0.001	**<0.001**	-0.760	0.000	**<0.001**	-1.657	0.000	**<0.001**
Depth scale												
multi.FD	-1.578	0.007	**<0.001**	-1.150	0.006	**<0.001**	-1.542	0.000	**<0.001**	-3.979	0.000	**<0.001**
LH	-0.800	0.007	**<0.001**	0.166	0.013	**<0.001**	-0.660	0.000	**<0.001**	-1.763	0.000	**<0.001**
SH	-0.827	0.005	**<0.001**	-0.730	0.006	**<0.001**	-1.231	0.000	**<0.001**	-3.050	0.000	**<0.001**
SLA	-0.592	0.006	**<0.001**	-0.422	0.004	**<0.001**	-0.853	0.000	**<0.001**	-3.289	0.000	**<0.001**
LDMC	-0.743	0.004	**<0.001**	-0.776	0.004	**<0.001**	-0.918	0.000	**<0.001**	-2.829	0.000	**<0.001**
FD	-0.753	0.005	**<0.001**	-0.469	0.005	**<0.001**	-0.754	0.000	**<0.001**	-2.770	0.000	**<0.001**
Lake scale												
multi.FD	-1.711	0.042	**<0.001**	-1.057	0.034	**<0.001**	-1.520	0.002	**<0.001**	-4.982	0.003	**<0.001**
LH	-0.667	0.043	**0.007**	0.407	0.053	0.495	-0.425	0.002	**0.015**	-2.369	0.003	**<0.001**
SH	-0.858	0.026	**<0.001**	-0.696	0.032	**0.001**	-1.404	0.001	**<0.001**	-3.489	0.002	**<0.001**
SLA	-0.610	0.035	**0.004**	-0.359	0.024	**0.006**	-0.657	0.002	**0.004**	-3.812	0.002	**<0.001**
LDMC	-0.733	0.024	**<0.001**	-0.737	0.020	**<0.001**	-1.000	0.001	**<0.001**	-3.501	0.002	**<0.001**
FD	-0.849	0.023	**<0.001**	-0.469	0.023	**<0.001**	-0.679	0.001	**<0.001**	-3.320	0.002	**<0.001**
Region scale												
multi.FD	-1.805	0.35		-0.541	0.035		-2.438	na		-1.577	na	
LH	-0.635	0.419		na	na		-1.103	na		na	na	
SH	-1.555	0.015		-0.606	0.055		-2.144	na		-0.879	na	
SLA	-0.187	0.273		0.117	0.229		-1.194	na		-0.777	na	
LDMC	-0.909	0.144		-0.713	0.076		-1.428	na		-1.155	na	
FD	-1.401	0.028		-0.593	0.042		-1.538	na		-1.243	na	

Table S4. Results of Wilcoxon signed-ranks test for metrics of functional alpha (standardized effect size of the pairwise trait distance, SES PW and standardized effect size of the nearest-neighbor trait distance, SES NN) and beta (standardized effect size of the pairwise trait dissimilarity, SES Dpw and standardized effect size of the nearest-neighbor trait dissimilarity, SES Dnn) dispersion at four studied ecological scales (i.e., plot, depth, lake and regional scale). The mean and standard errors of SESs are shown. The *P*-values at regional scale are not shown because there were only two groups (low and high elevation). Significant *P*-values are shown in boldface type. Abbreviations are: multi.FD indicates multiple traits metrics; LH indicates life history; SH indicates shoot height; SLA indicates specific leaf area; LDMC indicates leaf dry mass content; FD indicates flowering duration; na indicates not analysis.

Functional traits	Fixed effects	Random effects	*d.f.*	Estimate	SE	*t-value*	*LRT*	*P-value*
SES PW at depth scale								
multi.FD	depth	(1|region)+(1|lake)+(depth|plot)	8	0.026	0.029	0.907	0.792	0.374
LH	depth	(region|lake)+(depth|site)	9	-0.092	0.032	0.032	8.122	**0.004**
SH	depth	(1|region)+(1|lake)+(depth|plot)	8	0.063	0.021	3.051	8.612	**0.003**
SLA	depth	(1|region)+(1|lake)+(depth|plot)	8	-0.023	0.024	-0.932	0.833	0.361
LDMC	depth	(1|region)+(1|lake)+(depth|plot)	8	0.027	0.021	1.324	1.715	0.190
FD	depth	(1|region)+(1|lake)+(depth|plot)	8	0.104	0.022	4.816	21.060	**<0.001**
SES Dpw at depth scale								
multi.FD	depth	(1|region)+(1|lake)+(depth|plot)	8	-0.026	0.015	-1.708	2.899	0.089
LH	depth	(region|lake)+(depth|site)	9	-0.031	0.013	-2.411	5.772	**0.016**
SH	depth	(1|region)+(1|lake)+(depth|plot)	8	0.023	0.012	1.867	3.433	0.064
SLA	depth	(region|lake)+(depth|site)	9	-0.055	0.017	-3.171	9.592	**0.002**
LDMC	depth	(1|region)+(1|lake)+(depth|plot)	8	0.045	0.009	4.766	20.836	**<0.001**
FD	depth	(1|region)+(1|lake)+(depth|plot)	8	0.009	0.010	0.889	0.780	0.377
SES PW at lake scale								
multi.FD	lwt	(1 | region) + (1 | lake)	5	-0.658	1.036	-0.635	4.165	**0.041**
LH	lwt	(1 | region)	4	-0.704	1.076	-0.655	4.328	**0.038**
SH	lwt	(1 | region) + (1 | lake)	5	-0.112	0.978	-0.115	0.451	0.502
SLA	lwt	(1 | region) + (1 | lake)	5	-0.449	1.485	-0.302	5.523	**0.019**
LDMC	lwt	(1 | region) + (1 | lake)	5	-0.640	0.613	-1.044	6.353	**0.012**
FD	lwt	(1 | region) + (1 | lake)	5	0.270	0.762	0.355	2.158	0.142
SES Dpw at lake scale								
multi.FD	lwt	(1|region)+(lwt|lake)+(lake|depth)	10	-0.048	0.024	-1.971	4.224	**0.040**
LH	lwt	(1|region)	4	-0.055	0.023	-2.359	6.011	**0.014**
SH	lwt	(1|region)	4	-0.061	0.016	-3.877	14.452	**<0.001**
SLA	lwt	(1|region)+(lwt|lake)+(lake|depth)	10	-0.045	0.038	-1.191	0.921	0.337
LDMC	lwt	(1|region)	4	0.010	0.014	0.687	0.357	0.550
FD	lwt	(1|region)+(lwt|lake)+(lake|depth)	10	-0.031	0.053	-0.578	0.436	0.509
SES PW at regional scale								
multi.FD	elevation	(1 | lake)	4	-0.742	0.373	-1.987	3.656	0.056
LH	elevation	(1 | lake)	4	-0.841	0.376	-2.235	4.537	**0.033**
SH	elevation	(1 | lake)	4	-0.086	0.215	-0.401	0.160	0.689
SLA	elevation	(1 | lake)	4	-0.539	0.318	-1.698	2.721	0.099
LDMC	elevation	(1 | lake)	4	-0.656	0.175	-3.754	10.291	**0.001**
FD	elevation	(1 | lake)	4	0.348	0.209	1.664	2.622	0.105
SES Dpw at regional scale								
multi.FD	elevation	(1 | lake)	4	-0.448	0.163	-2.751	7.566	**0.012**
LH	elevation	(1 | lake)	4	-0.354	0.184	-1.924	3.444	0.063
SH	elevation	(1 | lake)	4	-0.082	0.103	-0.800	0.632	0.427
SLA	elevation	(1 | lake)	4	-0.642	0.278	-2.308	4.658	**0.031**

LDMC	elevation	(1 | lake)	4	-0.271	0.072	-3.743	11.033	**<0.001**
FD	elevation	(1 | lake)	4	-0.063	0.109	-0.575	0.328	0.567

Table S5. Results of the optimal linear mixed-effects models for the effects of major environmental gradients on the pairwise metrics of functional alpha (standardized effect size of the pairwise trait distance, SES PW) and beta (standardized effect size of the pairwise trait dissimilarity, SES Dpw) dispersion at specific ecological scales. Fixed effects structures, random effects structure, degrees of freedom (*d.f.*), slope (estimate) and its standard error (SE) of fixed effects, *t*-value, Likelihood ratio test (LRT) parameters and *P*-value of linear mixed-effects models are showed. Positive estimate indicates functional dispersion metrics increase along environmental gradients. Negative estimate indicates functional dispersion metrics decrease along environmental gradients. Significant *P*-values are shown in boldface type. Abbreviations are: multi.FD indicates multiple traits metrics; LH indicates life history; SH indicates shoot height; SLA indicates specific leaf area; LDMC indicates leaf dry mass content; FD indicates flowering duration; lwt indicates level of water transparency (Secchi depth, SD), and we separated the log-transformed SD (cm) gradients as five levels (1: 1.47-1.76; 2: 1.76-2.06; 3: 2.06-2.36; 4: 2.36-2.66; 5: 2.66-2.95).

Functional traits	Fixed effects	Random effects	*d.f.*	Estimate	SE	*t-value*	*L.Ratio*	*P*
SES NN at depth scale								
multi.FD	depth	(1|region)+(1|lake)+(depth|plot)	8	-0.025	0.026	-0.934	0.851	0.356
LH	depth	(region|lake)+(depth|site)	9	-0.248	0.046	-5.380	25.177	**<0.001**
SH	depth	(1|region)+(1|lake)+(depth|plot)	9	0.062	0.021	2.942	8.134	**0.004**
SLA	depth	(1|region)+(1|lake)+(depth|plot)	8	-0.070	0.024	-2.992	8.740	**0.003**
LDMC	depth	(1|region)+(1|lake)+(depth|plot)	8	0.021	0.019	1.124	1.221	0.269
FD	depth	(1|region)+(1|lake)+(depth|plot)	8	0.049	0.019	2.499	6.220	**0.013**
SES Dnn at depth scale								
multi.FD	depth	(1|region)+(1|lake)+(depth|plot)	8	0.181	0.023	7.718	42.902	**<0.001**
LH	depth	(1|region)+(1|lake)+(depth|plot)	8	0.227	0.029	7.749	48.014	**<0.001**
SH	depth	(1|region)+(1|lake)+(depth|plot)	8	0.157	0.018	8.497	53.552	**<0.001**
SLA	depth	(1|region)+(1|lake)+(depth|plot)	8	0.171	0.020	8.638	51.392	**<0.001**
LDMC	depth	(1|region)+(1|lake)+(depth|plot)	8	0.182	0.017	10.550	67.376	**<0.001**
FD	depth	(1|region)+(1|lake)+(depth|plot)	8	0.117	0.013	9.239	56.098	**<0.001**
SES NN at lake scale								
multi.FD	lwt	(1 | region) + (1 | lake)	5	0.036	0.099	0.366	0.051	0.821
LH	lwt	(1 | region)	4	0.102	0.118	0.868	0.763	0.383
SH	lwt	(1 | region) + (1 | lake)	5	-0.036	0.113	-0.317	0.115	0.735
SLA	lwt	(1 | region)	4	0.006	0.084	0.069	0.018	0.894
LDMC	lwt	(1 | region) + (1 | lake)	5	0.047	0.065	0.725	0.413	0.521
FD	lwt	(1 | region) + (1 | lake)	5	0.028	0.077	0.367	0.304	0.581
SES Dnn at lake scale								
multi.FD	lwt	(1|region)+(lwt|lake)+(lake|depth)	10	-0.361	0.143	-2.534	5.578	**0.018**
LH	lwt	(1|region)	4	-0.144	0.067	-2.143	3.869	**0.049**
SH	lwt	(1|region)	4	-0.245	0.044	-5.535	23.810	**<0.001**
SLA	lwt	(1|region)+(lwt|lake)+(lake|depth)	10	-0.233	0.070	-3.326	6.250	**0.012**
LDMC	lwt	(1|region)	4	-0.244	0.049	-4.950	19.101	**<0.001**
FD	lwt	(1|region)+(lwt|lake)+(lake|depth)	10	-0.244	0.088	-2.785	5.827	**0.016**
SES NN at regional scale								
multi.FD	elevation	(1 | lake)	4	-0.567	0.309	-1.831	3.140	0.076
LH	elevation	(1 | lake)	4	-0.209	0.538	-0.388	0.145	0.703
SH	elevation	(1 | lake)	4	0.303	0.302	1.005	0.989	0.320
SLA	elevation	(1 | lake)	4	-0.387	0.216	-1.790	3.006	0.083
LDMC	elevation	(1 | lake)	4	-0.529	0.156	-3.390	9.392	**0.002**
FD	elevation	(1 | lake)	4	0.488	0.203	2.405	5.183	**0.023**
SES Dnn at regional scale								
multi.FD	elevation	(1 | lake)	4	0.318	0.287	1.109	1.167	0.280
LH	elevation	(1 | lake)	4	0.401	0.351	1.143	1.262	0.261
SH	elevation	(1 | lake)	4	-0.093	0.210	-0.444	0.197	0.658
SLA	elevation	(1 | lake)	4	-0.008	0.308	-0.026	0.001	0.979
LDMC	elevation	(1 | lake)	4	-0.002	0.209	-0.011	0.000	0.992

FD	elevation	(1 | lake)	4	-0.324	0.285	-1.136	1.256	0.262

Table S6. Results of the optimal linear mixed-effects models for the effects of major environmental gradients on the nearest-neighbor metrics of functional alpha (standardized effect size of the nearest-neighbor trait distance, SES NN) and beta (standardized effect size of the nearest-neighbor trait dissimilarity, SES Dnn) dispersion at specific ecological scales. Fixed effects structures, random effects structure, degrees of freedom (*d.f.*), slope (estimate) and its standard error (SE) of fixed effects, *t*-value, Likelihood ratio test (LRT) parameters and *P*-value of linear mixed-effects models are showed. Negative estimate indicates functional dispersion metrics decrease along environmental gradients. Significant *P*-values are shown in boldface type. Abbreviations are: multi.FD indicates multiple traits metrics; LH indicates life history; SH indicates shoot height; SLA indicates specific leaf area; LDMC indicates leaf dry mass content; FD indicates flowering duration; lwt indicates level of water transparency (Secchi depth, SD), and we separated the log-transformed SD (cm) gradients as five levels (1: 1.47-1.76; 2: 1.76-2.06; 3: 2.06-2.36; 4: 2.36-2.66; 5: 2.66-2.95).

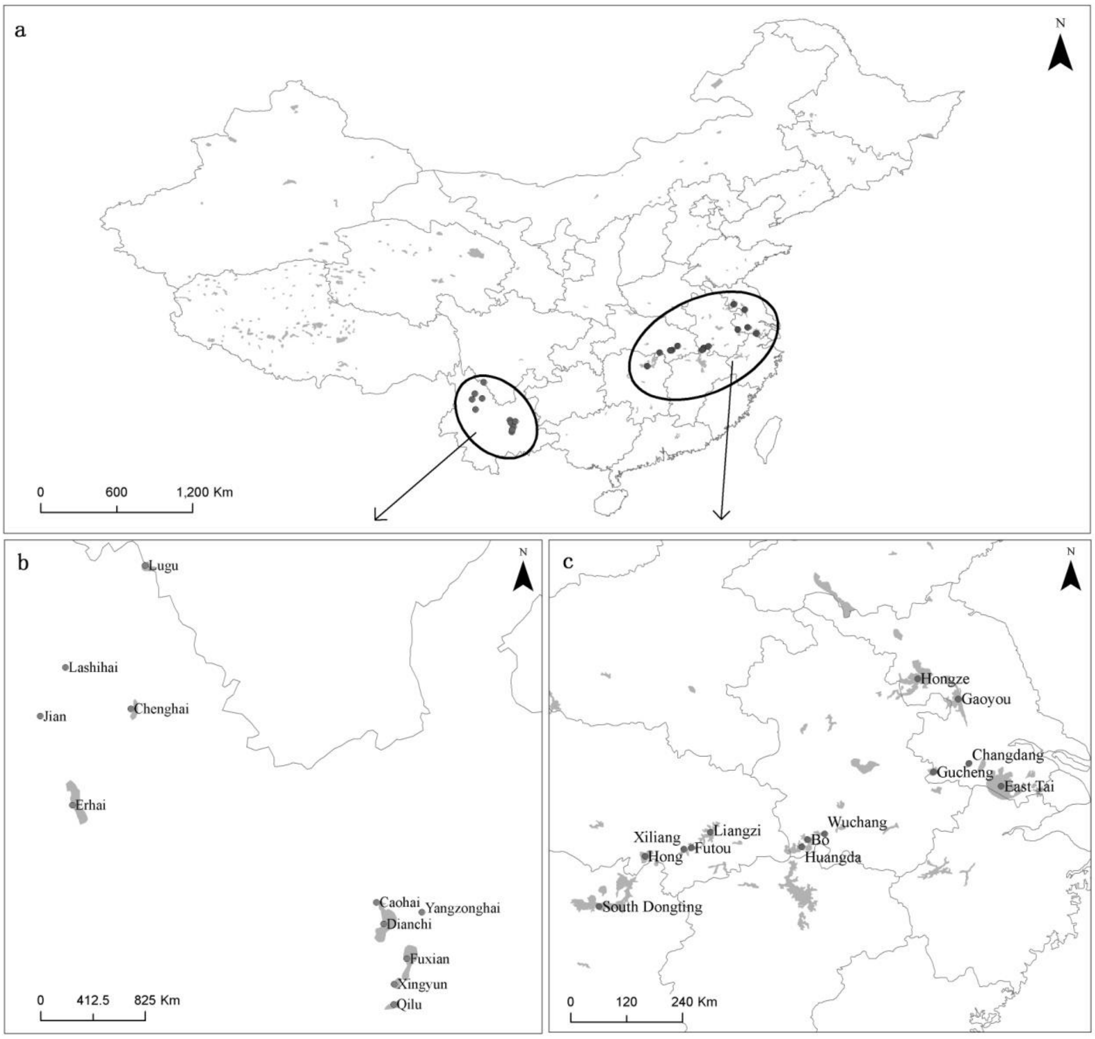

Figure S1. Geographical location and spatial distribution of studied lakes (black point) in south China (a), with 11 lakes in Yunnan-Guizhou plateau (b) and 13 lakes along the middle and lower reaches of the Yangtze River (c). Map of these lakes was drawn by ArcGIS 10.0 (http://www.esrichina-bj.cn/softwareproduct/ArcGIS/).

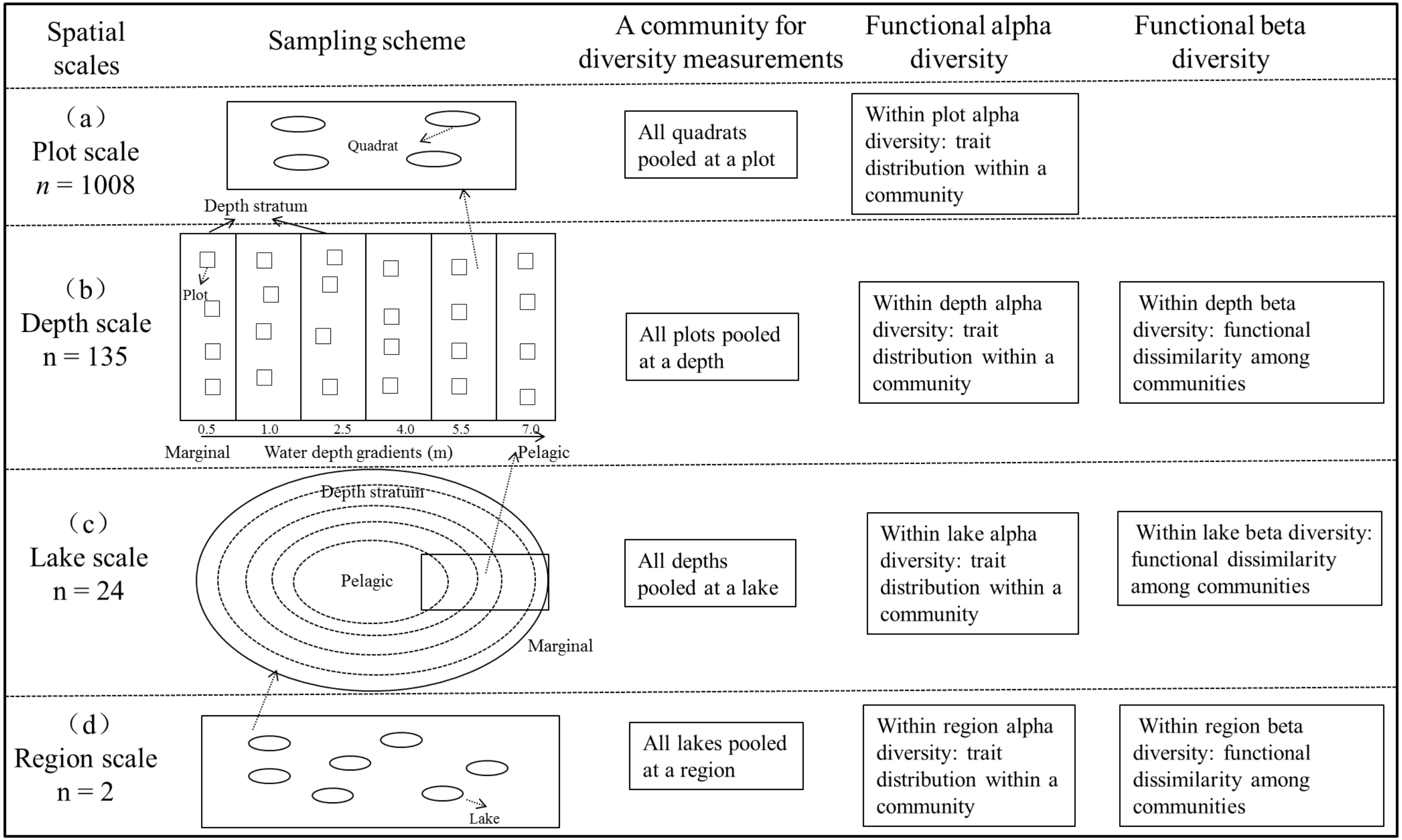

Spatial scales
Sampling scheme
A community for diversity measurements
Functional alpha diversity
Functional beta diversity
(a)
Plot scale
n = 1008
Quadrat
All quadrats pooled at a plot
Within plot alpha diversity: trait distribution within a community
Depth stratum
(b)
Depth scale
n = 135
Plot
0.5
1.0
2.5
4.0
5.5
7.0
Marginal
Water depth gradients (m)
Pelagic
All plots pooled at a depth
Within depth alpha diversity: trait distribution within a community
Within depth beta diversity: functional dissimilarity among communities
(c)
Lake scale
n = 24
Depth stratum
Pelagic
Marginal
All depths pooled at a lake
Within lake alpha diversity: trait distribution within a community
Within lake beta diversity: functional dissimilarity among communities
(d)
Region scale
n = 2
Lake
All lakes pooled at a region
Within region alpha diversity: trait distribution within a community
Within region beta diversity: functional dissimilarity among communities

Figure S2. The hierarchical nested sampling scheme of this study and calculations of functional alpha and beta diversity across spatial scales. The plots were nested within different depth strata, the depth strata were nested within different lakes, and the lakes were nested within different region. We defined ecological communities at four nested spatial scales. Firstly, the plot scale was defined as four quadrats pooled into a 25 m^2 plot (a). Secondly, the depth scale was defined as all 25 m^2 plots at the same water depth (b). Third, the lake scale was defined as all depths within the same lake (c). Fourth, the regional scale was defined as all lakes within one of two regions (d)

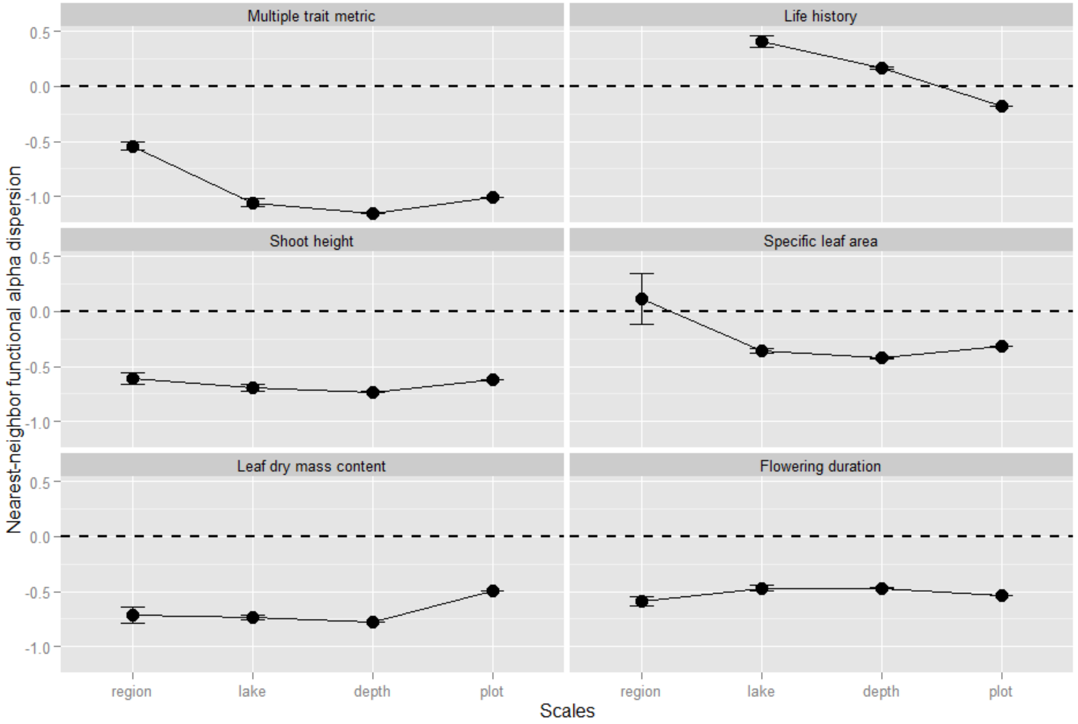

Figure S3. The effects of spatial scale on nearest-neighbor functional alpha dispersion. The values lower than 0 indicates a clustering of alpha diversity. The values higher than 0 indicate an over-dispersion of alpha diversity. Each point indicates the mean SES values for each trait metric of each scale.

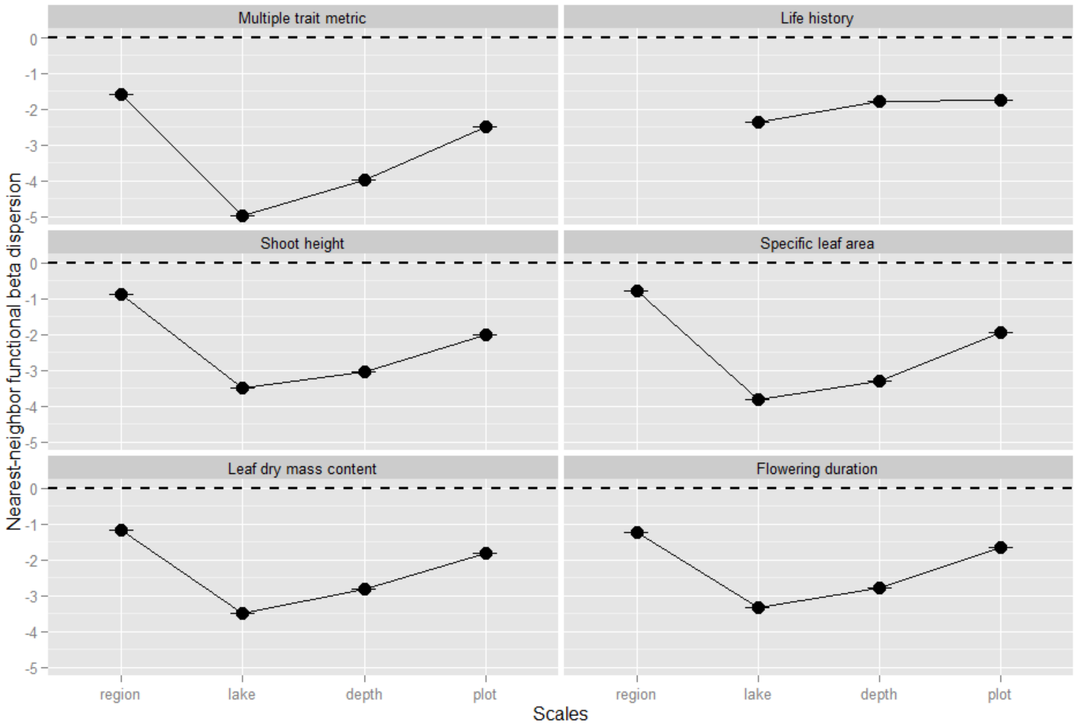

Figure S4. The effects of spatial scale on nearest-neighbor functional beta dispersion. The values lower than 0 indicate a clustering of beta diversity. The values higher than 0 indicate an over-dispersion of beta diversity. Each point indicates the mean SES values for each trait metric of each scale.

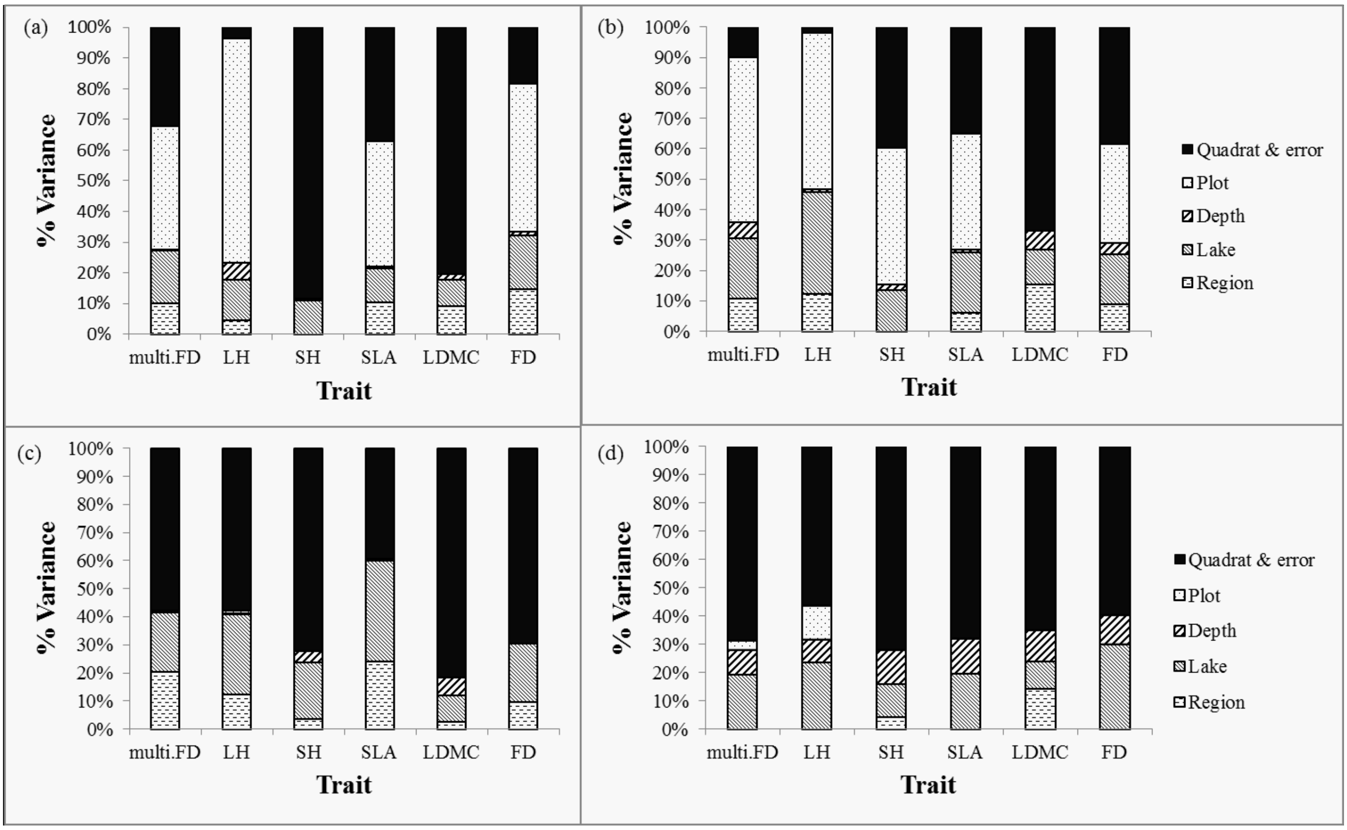

Figure S5. Variance partitioning of functional dispersion across spatial scales. (a) pairwise functional alpha diversity, (b) nearest-neighbor functional alpha dispersion, (c) pairwise functional beta diversity, (d) nearest-neighbor functional beta dispersion. Abbreviations are: multi.FD indicates multiple traits metrics; LH indicates life history; SH indicates shoot height; SLA indicates specific leaf area; LDMC indicates leaf dry mass content; FD indicates flowering duration.

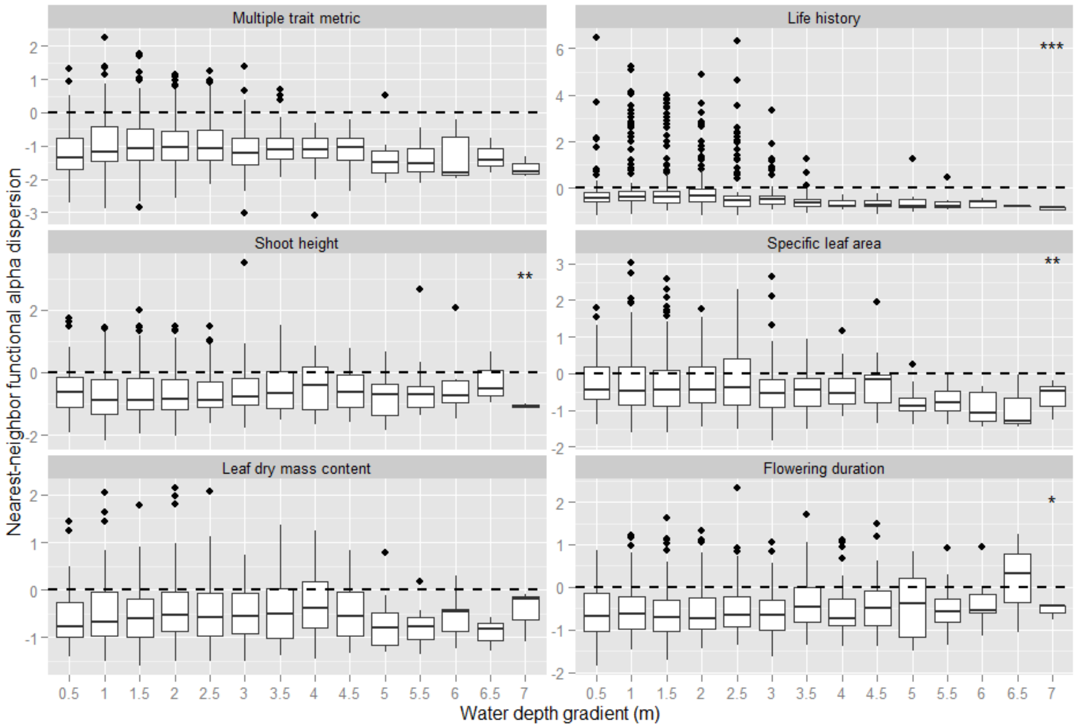

Figure S6. The effects of water depth gradient on nearest-neighbor functional alpha dispersion at the depth scale. The values lower than 0 indicate a clustering of alpha diversity. The values higher than 0 indicate an over-dispersion of alpha diversity. *: $p< 0.05$, **: $p< 0.01$, ***: $p< 0.001$.The *p*-values are the results of linear mixed effects models (see Table S6).

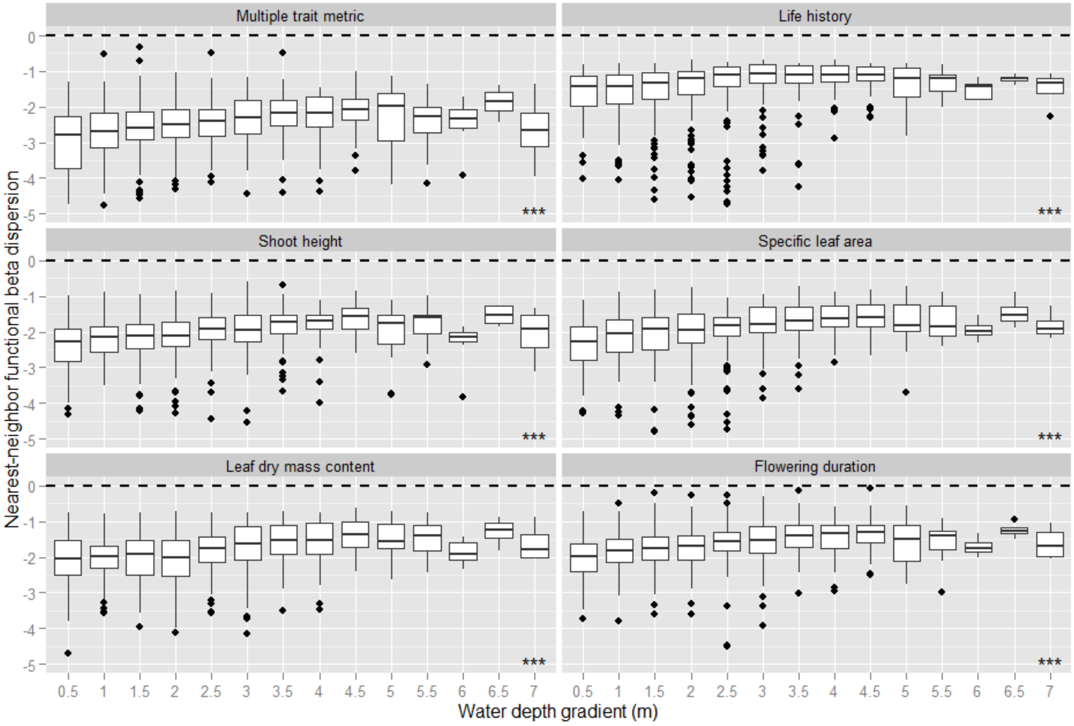

Figure S7. The effects of water depth gradient on nearest-neighbor functional beta dispersion at the depth scale. The values lower than 0 indicate a clustering of beta diversity. The values higher than 0 indicate an over-dispersion of beta diversity. *: $p< 0.05$, **: $p< 0.01$, ***: $p< 0.001$.The *p*-values are the results of linear mixed effects models (see Table S6).

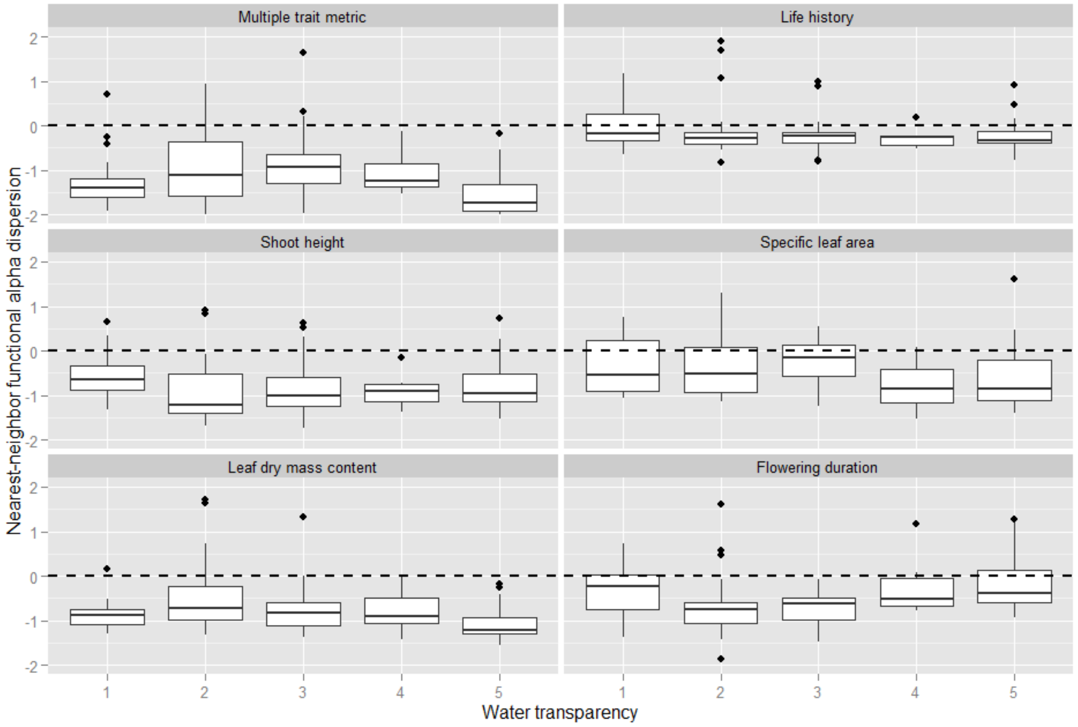

Figure S8. The effects of water transparency (Secchi depth, SD) on nearest-neighbor functional alpha dispersion at the lake scale. The values lower than 0 indicate a clustering of alpha diversity. The values higher than 0 indicate an over-dispersion of alpha diversity. We separated the log-transformed SD (cm) gradients as five levels (1: 1.47-1.76; 2: 1.76-2.06; 3: 2.06-2.36; 4: 2.36-2.66; 5: 2.66-2.95). *: $p< 0.05$, **: $p< 0.01$, ***: $p< 0.001$.The p-values are the results of linear mixed effects models (see Table S6).

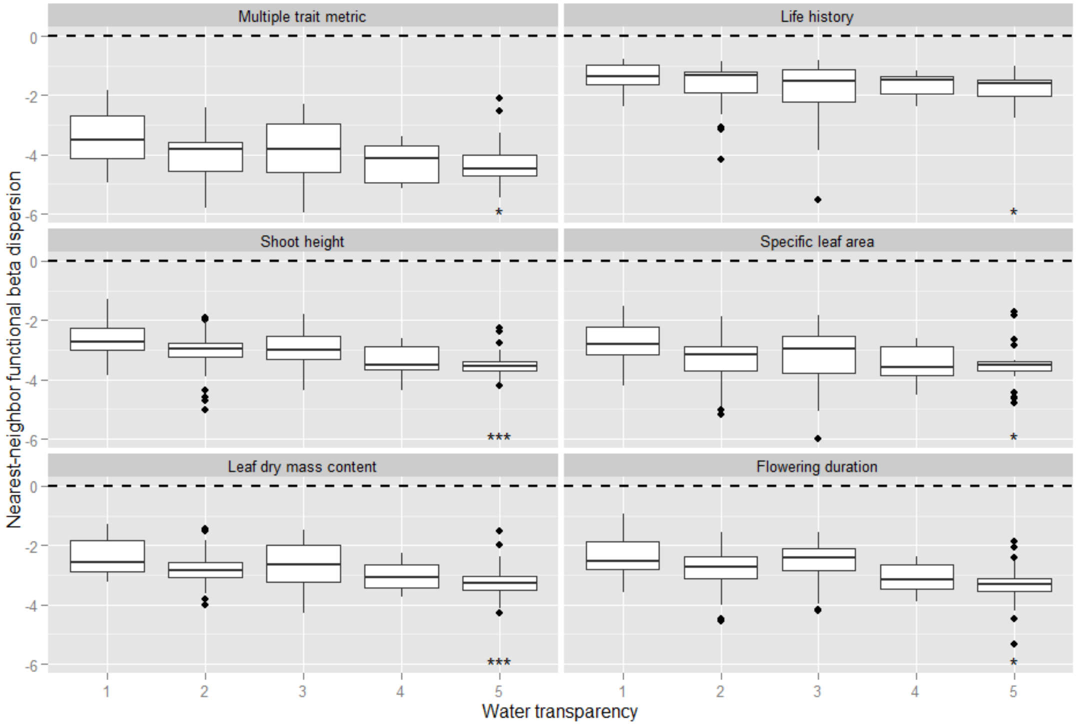

Figure S9. The effects of water transparency (Secchi depth, SD) on nearest-neighbor functional beta dispersion (standardized effect size of the nearest-neighbor trait dissimilarity, SES Dnn) at the lake scale. The values lower than 0 indicate a clustering of beta diversity. The values higher than 0 indicate an over-dispersion of beta diversity. We separated the log-transformed SD (cm) gradients as five levels (1: 1.47-1.76; 2: 1.76-2.06; 3: 2.06-2.36; 4: 2.36-2.66; 5: 2.66-2.95). *: $p< 0.05$, **: $p< 0.01$, ***: $p< 0.001$.The *p*-values are the results of linear mixed effects models (see Table S6).

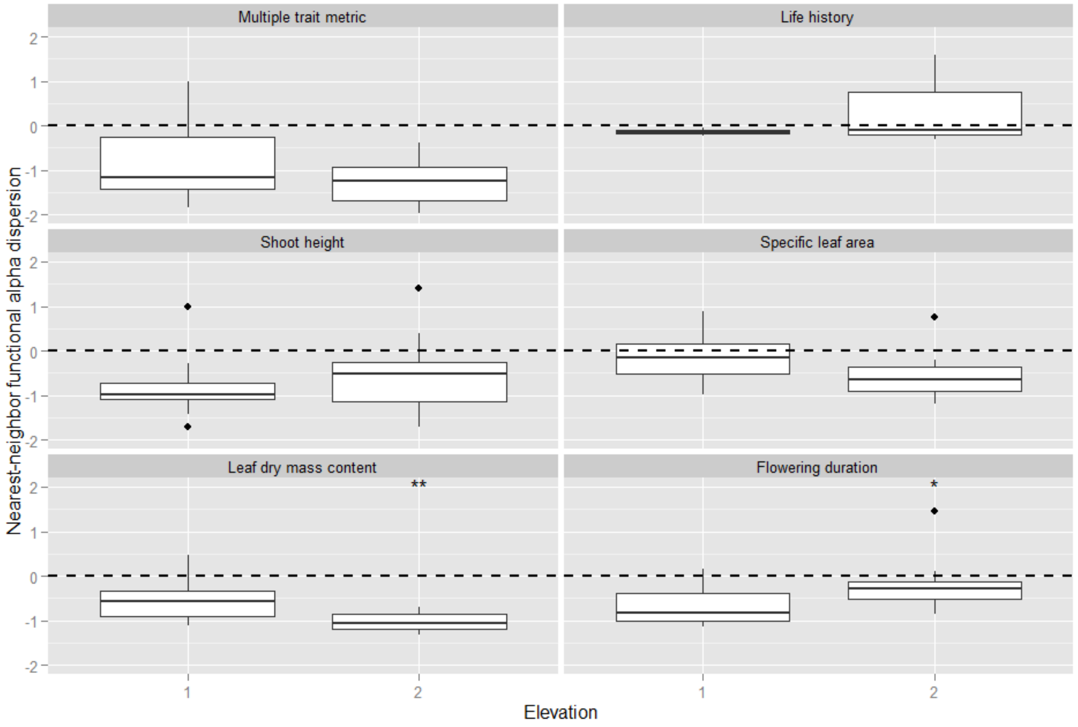

Figure S10. The effects of elevation (1, lakes with low elevation at the middle and lower reaches of the Yangtze River, 16.3 m above sea level; 2, lakes with high elevation at Yunnan-guizhou plateau, 1967.4 m above sea level) on nearest-neighbor functional alpha dispersion (standardized effect size of the nearest-neighbor trait distance, SES NN) at the regional scale. The values lower than 0 indicate a clustering of alpha diversity. The values higher than 0 indicate an over-dispersion of alpha diversity. *: $p< 0.05$, **: $p< 0.01$, ***: $p< 0.001$.The *p*-values are the results of linear mixed effects models (see Table S6).

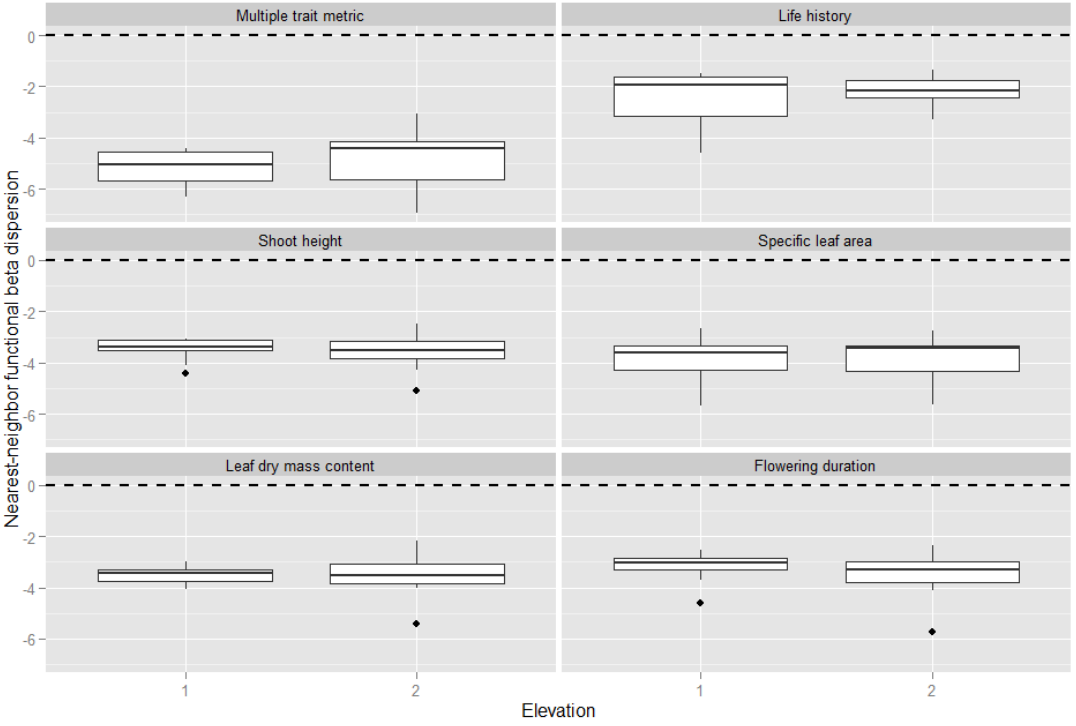

Figure S11. The effects of elevation (1, lakes with low elevation at the middle and lower reaches of the Yangtze River, 16.3 m above sea level; 2, lakes with high elevation at Yunnan-guizhou plateau, 1967.4 m above sea level) on nearest-neighbor functional beta dispersion (standardized effect size of the nearest-neighbor trait dissimilarity, SES Dnn) at the regional scale. The values lower than 0 indicate a clustering of beta diversity. The values higher than 0 indicate an over-dispersion of beta diversity. *: $p< 0.05$, **: $p< 0.01$, ***: $p< 0.001$.The *p*-values are the results of linear mixed effects models (see Table S6).
